# Supplementary material for: Competitiveness of Xinjiang’s mutton industry based on diamond model
Source: PLoS One. 2021 Oct 28;16(10):e0257669. doi: 10.1371/journal.pone.0257669 (PMC8553171; doi:10.1371/journal.pone.0257669)
Supplement: Attachment — Submitted filename: renamed_78649.doc [file pone.0257669.s001.doc]

**Response to reviewers’ comments on PONE-D-21-00814**

Dear editor of Plos One:

Thank you very much for your detailed evaluations and suggestions!

Your comments were highly insightful and enabled us to greatly improve the quality of our paper. We have carefully revised our manuscript entitled by "**Research on the Competitiveness of the Xinjiang Mutton Industry based on Diamond Model**" according to your advice. Additionally, the editorial errors throughout the manuscript have been reedited and rectified. We wish the revision could meet with your approval.

Here, we listed the point-by-point responses to your detailed comments and suggestions (With blue). As follows:

1. Line 4: I have changed the name (Dawut Ablat) to (Ablat Dawut), cause in our language first name put first, and then put family name.
2. Line 186-193: I have further clarified the evaluation index and analysis method of qualitative and quantitative analysis in this method section.
3. Line 204: The data sources are from China Statistical Yearbook, China animal husbandry and veterinary Yearbook and other legal databases. The authenticity of the data is evaluated by correlation and principal component analysis.
4. Line 205-209: I have defined the data sources in this section.

5. Line 217: In the aspect of analysis method, the software and analysis method are defined, and added Fig 5 and 6.

6. Line 306: The structure of this paragraph has been adjusted accordingly.

7. Line 309: Here, i have corrected the unit of data.

8. Line 310: Here, i have corrected the unit of data.

9. Line 453-468: I have reanalyzed this section.

10. Line 469-488: I have reanalyzed this section.

11. Line 490: I have added new content and deleted the redundant part in this section on the basis of the original.
